# Unravelling morphoea aetiopathogenesis by next-generation sequencing of paired skin biopsies

**DOI:** 10.1007/s00403-023-02541-5

**Published:** 2023-03-13

**Authors:** Amanda M. Saracino, Daniel Kelberman, Georg W. Otto, Andrey Gagunashvili, David J. Abraham, Christopher P. Denton

**Affiliations:** 1 Division of Medicine, Centre for Rheumatology and Connective Tissues Diseases, University College London, London, UK; 2 Department of Dermatology, Royal Free NHS Foundation Trust, London, UK; 3 GOSgene, Genetics and Genomic Medicine, Great Ormand Street Institute of Child Health, University College London, London, UK; 4 Department of Rheumatology, Royal Free NHS Foundation Trust, London, UK; 5 Melbourne Dermatology Clinic, 258 Park Street, South Melbourne, VIC 3205 Australia

**Keywords:** Morphoea, Linear morphoea, Localised scleroderma, Aetiopathogenesis, Next-generation sequencing, Genomics, Transcriptomics, Gene expression

## Abstract

**Background:**

Morphoea can have a significant disease burden. Aetiopathogenesis remains poorly understood, with very limited existing genetic studies. Linear morphoea (LM) may follow Blascho’s lines of epidermal development, providing potential pathogenic clues.

**Objective:**

The first objective of this study was to identify the presence of primary somatic epidermal mosaicism in LM. The second objective was tTo explore differential gene expression in morphoea epidermis and dermis to identify potential pathogenic molecular pathways and tissue layer cross-talk.

**Methodology:**

Skin biopsies from paired affected and contralateral unaffected skin were taken from 16 patients with LM. Epidermis and dermis were isolated using a 2-step chemical-physical separation protocol. Whole Genome Sequencing (WGS; *n* = 4 epidermal) and RNA-seq (*n* = 5-epidermal, *n* = 5-dermal) with gene expression analysis via GSEA-MSigDBv6.3 and PANTHER-v14.1 pathway analyses, were performed. RTqPCR and immunohistochemistry were used to replicate key results.

**Results:**

Sixteen participants (93.8% female, mean age 27.7 yrs disease-onset) were included. Epidermal WGS identified no single affected gene or SNV. However, many potential disease-relevant pathogenic variants were present, including ADAMTSL1 and ADAMTS16. A highly proliferative, inflammatory and profibrotic epidermis was seen, with significantly-overexpressed TNFα-via-NFkB, TGFβ, IL6/JAKSTAT and IFN-signaling, apoptosis, p53 and KRAS-responses. Upregulated IFI27 and downregulated LAMA4 potentially represent initiating epidermal ‘damage’ signals and enhanced epidermal-dermal communication. Morphoea dermis exhibited significant profibrotic, B-cell and IFN-signatures, and upregulated morphogenic patterning pathways such as Wnt.

**Conclusion:**

This study supports the absence of somatic epidermal mosaicism in LM, and identifies potential disease-driving epidermal mechanisms, epidermal-dermal interactions and disease-specific dermal differential-gene-expression in morphoea. We propose a potential molecular narrative for morphoea aetiopathogenesis which could help guide future targeted studies and therapies.

**Supplementary Information:**

The online version contains supplementary material available at 10.1007/s00403-023-02541-5.

## Introduction

Morphoea is characterised by fibrosis of the skin and/or underlying connective tissues, with the potential for significant functional and psychological impact. It is suggested that environmental triggers [1–3], occurring in a genetically susceptible individual, underpin the inflammation and deregulated tissue injury response in morphoea [4]. However, precise genetic susceptibility factors, inciting and propagating molecular mechanisms, remain unclear.

Linear morphoea (LM) may follow Blaschko’s lines of epidermal development, and hence may represent epidermal somatic mosaicism for a mutation conferring increased risk of disease at specific sites [5–9]. Accordingly, keratinocyte-derived signals and epidermal-dermal communication pathways vital to normal skin development and wound repair, are also key to pathological skin fibrosis and highly active, proliferative keratinocytes are seen in systemic sclerosis (SSc) [4, 10, 11].

However, LM is a non-congenital and morphologically heterogeneous dermal pathology, potentially suggesting more complex underlying aetiopathogenic mechanisms. Correspondingly, non-linear morphoea subtypes show alternative, but often symmetrical and somewhat predictably patterned skin involvement. As such, dermal fibroblasts have site-specific gene expression, known as positional identity (PI). Many molecular pathways instrumental in developmental patterning, regional-specific mesenchymal differentiation and epidermal fate, such as FGFs, TGF-β and Wnt [12, 13], are also involved in pathogenic fibrosis and SSc [14, 15]. Similarly, morphogenic and epidermal–dermal signaling pathways, including Wnt, Hedgehog [14, 16] and Notch [14, 17, 18], are deregulated in fibrosis and SSc [17, 19–23].

Morphoea’s morphological heterogeneity, clinical symmetry, patterning and possibly Blaschkoid distribution, may therefore provide clinical clues to potential underlying epidermal and dermal genetic aetiopathogenic and disease-driving mechanisms [4].

The goals of this study were to identify the presence or absence of primary somatic epidermal genomic variation (as a common single nucleotide variant (SNV), or differing SNVs in a commonly affected gene, across all study samples) in LM, and to explore differential gene expression (DGE) in isolated epidermal and dermal site-matched tissue pairs, to identify potential inciting and pathogenic pathways in the epidermis and dermis. We aimed to correlate our data with the very limited current genetic data in morphoea, to propose a possible genetic and molecular narrative underlying morphoea aetiopathogenesis and hence identify potential future study and therapeutic targets.

## Methodology

This study was approved by the National Research Ethics Service (London-Hampstead, MREC Reference 6398). Tissue specimens were obtained with written informed consent as part of an ongoing programme of research into the pathogenesis of scleroderma.

### Specimen source

Patients with LM involving the limb(s) and/or trunk identified from our previously characterised morphoea cohort were eligible for specimen collection [24]. A total of 16 patients were enrolled (Table 1). Details regarding sample selection for each molecular (DNA/RNA) and tissue layer (epidermal/dermal) dataset are described in the Supplemental Methods section.

**Table 1:** Study cohort; experimental studies and clinical characteristic

Study no	Sex, age onset (yrs)	Epidermal WGS	Epidermal/dermal RNA-seq	Validation studies	Disease status	Biopsy site activity	Site and phenotype biopsied	Cutaneous symptoms	Current treatment
1	F, 26	Yes	Epidermal*, dermal	Epidermal RT-qPCR	Stable	Yes	Upper limb; inflammatory, sclerotic	Pruritus, tingling	Topical
2	F, 18		Epidermal		Stable	No	Lower limb; inflammatory, sclerotic	Pruritus	Systemic
3	F, 19	Yes	Epidermal*, dermal	Epidermal RT-qPCR	Active	Yes	Upper limb; inflammatory	Pruritus	Topical
4	F, 19	Yes	Epidermal, dermal		Active	Yes	Upper limb; inflammatory, sclerotic	Tingling	Systemic
5	F, 51		Epidermal		Active	No	Lower limb; atrophic, pigmented	Nil	Nil; treatment naive
6	F, 32		Epidermal		Stable	No	Lower limb; atrophic, pigmented	Pain	Systemic
7	F, 21	Yes; failed sequencing	Epidermal, dermal		Active	Yes	Upper limb; inflammatory, sclerotic	Pruritus, pain	Systemic
8	F, 29	Yes	Epidermal*, dermal	Epidermal RT-qPCR	Active	Yes	Upper limb; inflammatory	Tingling	Systemic
9	F, 54			Epidermal RT-qPCR	Remission	No	Trunk; atrophic, pigmented	Nil	Nil; previous systemic
10	F, 26			Epidermal RT-qPCR	Remission	No	Lower limb; atrophic, pigmented	Pain	Nil, previous topical and systemic
11	F, 45			Epidermal RT-qPCR	Remission	No	Lower limb; atrophic, pigmented	Tingling	Nil, previous systemic
12	F, 12			Whole skin IHC	Active	Yes	Lower limb; pigmented	Pruritus	Topical, systemic
13	M, 8			Whole skin IHC	Stable	No	Sclerotic	Pain	Systemic
14	F, 10			Whole skin IHC	Active	Yes	Upper limb; sclerotic, pigmented	Nil	Systemic
15	F, 32			Whole skin IHC	Active	Yes	Lower limb; pigmented	Pruritus	Topical, systemic
16	F, 14			Whole skin IHC	Active	Yes	Trunk; sclerotic	pruritus, tingling	Topical, systemic

*Failed quality control with Beijing Genomics Institute for RNA-seq, alternative epidermal samples for RNA-seq selected (Study No. 2, 5 and 6)

Paired 4 mm whole skin punch-biopsies were taken from each participant; one or two from morphoea affected (lesional) skin, and one or two from site-matched contralateral unaffected skin. For tissues samples utilised for DNA/RNA isolation, epidermis was immediately chemically separated from the dermis utilising 3.8% ammonium thiocyanate (Sigma-Alrich USA) in Dulbecco's phosphate-buffered saline pH 7.4 at room temperature for 25 min. Residual epidermal tissue was gently curetted off the superficial dermal surface using a scalpel blade (no. 15) [25].

### DNA isolation, whole genome sequencing and analysis; epidermis

**Fig. 1:**
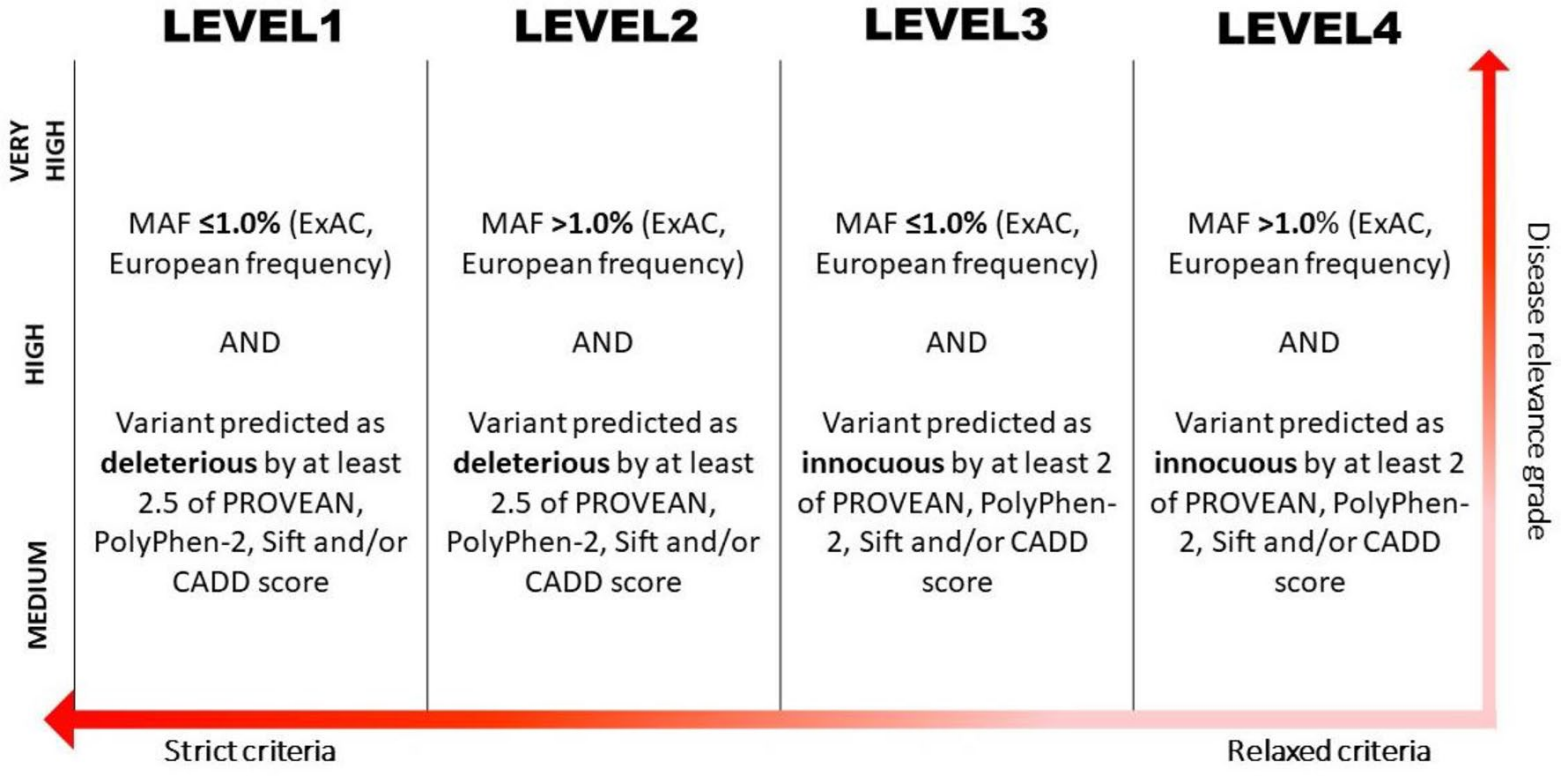
Classification strategy for disease relevant gene candidates (graded as very high, high or medium according to functional relevance to morphoea aetiopathogenesis; vertical grading) and for pathogenicity (according to allele frequency and pathogenicity criteria; horizontal classification ranking)

DNA was isolated from paired epidermal tissue and four selected paired samples underwent WGS. All identified genes with SNVs underwent network analysis utilising STRING online database (v11). Identified SNVs were then classified; graded according to disease relevance and sub-classified according to MAF (using ExAC) and pathogenicity (according to PolyPhen-2, PROVEAN, SIFT and CADD scores) (Supplemental Methods and Fig. 1).

### RNA isolation, sequencing and analysis; epidermis and dermis

Total RNA was isolated from paired epidermal and dermal tissue, and selected samples underwent RNA-seq. Epidermal and dermal differentially expressed genes (DEG) were further analysed via Gene Set Enrichment Analysis (GSEA), using MSigDB Hallmark gene sets [26, 27]. Enrichment was reported as significant if the false discovery rate (FDR) was less than 0.25 [28] and each GSEA set was ranked according to log2 fold change (log2FC).

For dermal RNA-seq data, further complimentary analysis via PANTHER (PANTHER Gene Ontology (GO)-Slim Biological Process) [29] was completed. An adjusted *P*-value was calculated using Bonferroni correction, with a statistical significance cut-off of < 0.05. STRING database was utilised to review protein–protein interactions between products of particular DEGs of interest. (Supplemental Methods).

### RT-qPCR and IHC of selected epidermal and dermal gene candidates derived from epidermal RNA-seq

Details can be found in the relevant Supplemental Methods sections.

## Results

### Epidermal protein coding single nucleotide variants

861 SNVs were identified in morphoea-affected epidermis, but absent in paired unaffected epidermis. Of these, 119 were protein-coding exonic and 72 nonsynonymous. No single common SNV or commonly affected gene was identified across all four sequenced epidermal tissue pairs.

A number of nonsynonymous protein-coding SNVs had high CADD scores (> 20) and pathogenicity rated as damaging or possibly damaging by at least two of PolyPhen-2, PROVEAN and SIFT algorithms, including; *ADAMTS16, ADAMTSL1* and *CBX2* (Table 2). STRING network analyses of these variants yielded no noteworthy gene clusters.

**Table 2:** All protein coding, nonsynonymous genomic variants (alphabetised, CADD scores rounded to the nearest whole number)

Gene symbol	Variant	Study participant	PolyPhen- 2	PolyPhen-2	PROVEAN	PROVEAN	SIFT	SIFT	CADD score	ExAC European frequency (%)
*ADAMTS16*	p.C1206V	4	0.999	Damaging	− 9.08	Damaging	0	Damaging	30	0
*ADAMTSL1*	p.A322T	3	0.999	Damaging	− 2.78	Damaging	0.005	Damaging	33	0
*C6orf15*	p.R27Q	8	0.955	Damaging	− 3.71	Damaging	0	Damaging	26	0.001522
*CACNA1D*	p.K776R, p.K796R	1	0.023	Benign	− 1.91	Neutral	0.064	Tolerated	24	0
*CAD*	p.E1420K, p.E1483K	1	0.190	Benign	− 2.58	Damaging	0.197	Tolerated	27	0
*CBX2*	p.G367R	8	0.561	Possibly damaging	− 2.21	Neutral	0	Damaging	24	0
*CBX2*	p.G367E	8	0.360	Benign	− 1.98	Neutral	0	Damaging	22	0
*CNTNAP3*	p.G1195R	3	0.999	Damaging	− 5.11	Damaging	0.015	Damaging	23	0
*CNTNAP3B*	p.D281H	1	N/A	NA	N/A	N/A	N/A	N/A	N/A	0
*CNTNAP3B*	p.V275I	1	0.191	Benign	− 0.80	Neutral	0.020	Damaging	6	0
*DEF8*	p.P71L, p.P131L, p.P121L, p.P192L	1	0.264	Benign	− 1.57	Neutral	0.262	Tolerated	24	0
*DEF8*	p.P71S, p.P131S, p.P121S, p.P192S	1	0.692	Possibly damaging	− 2.60	Damaging	0.029	Damaging	25	0
*DENND1C*	p.R515H	3	0.001	Benign	− 0.43	Neutral	0.545	Tolerated	7	0
*EFCC1*	p.A165T	4	0.118	Benign	N/A	N/A	N/A	N/A	22	0
*FAM186A*	p.T1377P	3	0	Benign	2.78	Neutral	1.000	Tolerated	< 1	0
*FAM231B*	p.S38T	3	N/A	N/A	N/A	N/A	N/A	N/A	< 1	2.20
*FAN1*	p.F866S	4	0.007	Benign	− 0.79	Neutral	0.457	Tolerated	3	0
*GOLGA6B*	p.G648D	3	0.017	Benign	1.40	Neutral	1.000	Tolerated	1	0
*HCFC1*	p.A934T	8	0.995	Damaging	− 1.68	Neutral	0.001	Damaging	32	0.0021
*HES6*	p.R49Q	8	0.737	Possibly damaging	− 2.68	Damaging	0.008	Damaging	24	0
*HRNR*	p.L1722S	8	0	Benign	1.80	Neutral	0.125	Tolerated	2	0.11
*HS6ST1*	p.V114G	3	0.679	Possibly damaging	0.32	Neutral	0.262	Tolerated	23	26.27
*IGSF3*	p.660Q, p.R680Q	8	0.345	Benign	− 1.71	Neutral	0.095	Tolerated	16	4.97
*IMPG2*	p.G2386A	4	0.109	Benign	− 1.73	Neutral	0.01	Damaging	20	0
*KIF21B*	p.R1371W, p.R1384W	1	0.993	Damaging	− 5.51	Damaging	0.001	Damaging	33	0
*KRT8*	p.S31A, p.S59A	8	0.001	Benign	0.27	Neutral	1.000	Tolerated	< 1	0.03
*MST1L*	p.R483C	3	N/A	N/A	N/A	N/A	N/A	N/A	N/A	0.02
*MUC12*	p.T3428I	8	N/A	N/A	− 2.00	Neutral	0.006	Damaging	4	0
*MUC20*	p.S182G	8	0.475	Possibly damaging	− 0.97	Neutral	0.411	Tolerated	< 1	0.03
*MUC4*	p.I2761V	4	0.001	Benign	− 0.12	Neutral	1.000	Tolerated	< 1	4.30
*MUC5B*	p.M2869T	1	0	Benign	1.03	Neutral	1.000	Tolerated	< 1	15.68
*NBPF20*	p.D3013E	4	N/A	N/A	N/A	N/A	N/A	N/A	< 1	0
*NDST2*	p.R464C	4	0.969	Damaging	− 5.99	Damaging	0.001	Damaging	32	0.0015
*NOS1AP*	p.A31V, p.A321V, p.A326V	3	0.511	Possibly damaging	− 2.35	Neutral	0.017	Damaging	30	0.00301
*NR2F2*	p.Y179S, p.Y159S, p.Y312S	4	0.944	Damaging	− 7.31	Damaging	0	Damaging	24	0
*OR11H12*	p.W68R	8	0	Benign	2.67	Neutral	0.475	Tolerated	< 1	0.001648
*OR2T6*	p.G151S	1	0.971	Damaging	− 1.61	Neutral	0.032	Damaging	23	0.001502
*PACS1*	p.Q35P	8	N/A	N/A	0.02	Neutral	0.364	Tolerated	< 1	0.06
*PARG*	p.R377W, p.R403W, p.R485W	8	N/A	N/A	N/A	N/A	N/A	N/A	N/A	N/A
*PAX2*	p.Q255R, p.Q286R, p.Q278R	4	0.104	Benign	− 1.49	Neutral	0.149	Tolerated	14	0
*PAX3*	p.G15D	1	0.025	Benign	− 1.37	Neutral	0.002	Damaging	24	0
*PAX3*	p.G15D	1	0.001	Benign	0.05	Neutral	0.251	Tolerated	22	0.006088
*PRAMEF10*	p.N459T	3	0	Benign	− 1.45	Neutral	0.254	Tolerated	< 1	0.007823
*PRAMEF6*	p.N381T	8	0	Benign	1.42	Neutral	1.000	Tolerated	< 1	0
*PRAMEF6*	p.S375N	8	0.996	Damaging	− 2.22	Neutral	0.017	Damaging	4	0
*PRDM9*	p.T713R	4	0.513	Possibly damaging	− 4.51	Damaging	0.001	Damaging	23	0.001675
*RFPL4A*	p.V179E	1	0.018	Benign	1.37	Neutral	0.910	Tolerated	< 1	17.13
*RGPD5;RGPD8*	p.R952S	3	0	Benign	2.33	Neutral	1.000	Tolerated	< 1	0
*RMDN3*	p.K285R	1	N/A	N/A	N/A	N/A	N/A	N/A	N/A	N/A
*RYR1*	p.D1377E	3	0.231	Benign	− 2.08	Neutral	0.193	Tolerated	23	0
*SAA2;SAA2-SAA4*	p.S156	8	0	Benign	2.70	Neutral	1.000	Tolerated	5	0
*SDR39U1*	p.D115Y, p.D89Y, p.D197Y	8	1.000	Damaging	− 8.85	Damaging	0	Damaging	32	0
*SGIP1*	p.G427R, p.G431R	8	0.999	Damaging	− 2.06	Neutral	0.031	Damaging	25	0
*SLC17A7*	p.F8V	1	0.002	Benign	− 0.53	Neutral	0.610	Tolerated	14	0
*SMG1*	p.I612K	8	0	Benign	− 3.00	Damaging	0.028	Damaging	17	3.57
*SPATA31D1*	p.A192P	3	0	Benign	3.61	Neutral	1.000	Tolerated	< 1	0.004496
*SPTBN1*	p.R1741H, p.R1754H	3	0.987	Damaging	− 4.60	Damaging	0.001	Damaging	31	0
*SYNE1*	p.V5268I, p.V5339I	1	0.006	Benign	0.36	Neutral	1.000	Tolerated	13	0
*TBC1D3B;TBC1D3D;TBC1D3G;TBC1D3H;TBC1D3I;TBC1D3L*	p.I117T	3	0.349	Benign	N/A	N/A	N/A	N/A	9	0
*TBC1D3D;TBC1D3H;TBC1D3I*	p.R399W	3	0	Benign	N/A	N/A	N/A	N/A	12	0
*TCP10*	p.A256S	1	0	Benign	0.69	Neutral	0.807	Tolerated	< 1	5.39
*TCP10*	p.R262W	8	0.035	Benign	0.52	Neutral	0.078	Tolerated	12	0.66
*TNS3*	p.S120Y	8	0.997	Damaging	− 3.34	Damaging	0	Damaging	31	0
*UFSP2*	p.E440K	8	0.074	Benign	− 1.13	Neutral	0.244	Tolerated	24	0
*URB1*	p.H967Y	1	0.469	Possibly damaging	− 0.49	Neutral	0.050	Damaging	25	0
*USP22*	p.F428S	3	0.998	Damaging	− 7.53	Damaging	0	Damaging	34	0
*WWC3*	pQ827K	1	0.108	Benign	− 0.73	Neutral	0.421	Tolerated	19	0
*ZNF608*	p.S1287L	1	0.716	Possibly damaging	− 1.31	Neutral	0.011	Damaging	23	0.001498
*ZNF614*	p.I201T	3	0.005	Benign	− 1.24	Neutral	0.275	Tolerated	< 1	0
*ZNF705E*	p.Q67R	4	N/A	N/A	N/A	N/A	N/A	N/A	N/A	0
*ZNF862*	p.R923K	4	0.001	Benign	0.35	Neutral	0.463	Tolerated	< 1	0
*ZP3*	p.s264P	4	0	Benign	0.71	Neutral	1.000	Tolerated	< 1	54.05

### Disease relevance of epidermal genomic variants

No protein coding nonsynonymous SNVs were graded as very high for disease relevance. Variants in the genes *ADAMTS16* and *ADAMTSL1* were graded as high for disease relevance and Level 1 for potential pathogenicity and rarity. All other protein-coding nonsynonymous variants were graded as medium disease relevance (Table 3).

**Table 3:** Potential gene candidates from epidermal whole genome sequencing as selected by network analyses and disease relevance; graded by potential relevance to morphea pathogenesis, and sub-categorised by Level, based on potential pathogenicity

Disease/functional relevance grade	Level 1	Level 2	Level 3	Level 4	Non-coding variants
Very high					*CCL5, FGF9, HBEGF, SMAD4, SMAD6*
High	*ADAMTS16, ADAMTSL1*				*ACTN4, ADAM9, ADAMTS14, ADAMTS6, DTX2, FLRT2, ITGB1, LTBP1, MAP3K7, MAP3K13, MTOR, NANOG, NFE2L2, PIAS1, PIK3CA, POU5F1, PTEN, RB1CC1, ROCK1, SPRTN*
Medium	C6orf15, CBX2 (p.G367R), HES6, CNTNAP3, DEF8*, HCFC1, NDST2, NOS1AP, NR2F2, OR2T6, PRDM9, SDR39U1, SGIP1, SMG1, SPTBN1, TNS3, URB1, USP22, ZNF608		*CAD, CBX2 (G367E), CNTNAP3B, DEF8*^*^*^*, DENND1C, EFCC1, FAM186A, FAN1, GOGLA6B, HRNR, MUC4, MUC20, NBPF20, OR11H12, PACS1, PARG, PAX2, PAX3, PRAMEF10, PRAMEF6, RGPDS;RGPD8, RYR1, SAA2;SAA2-SAA4, SLC17A7, SPATA31D1, SYNE1, TBC1D3B;TBC1D3D;TBC1D3G;TBC1D3H;TBC1D3I;TBC1D3L, TBC1D3D;TBC1D3H;TBC1D3I, WWC3, ZNF614, ZNF705E, ZNF862*	*FAM231B, HS6ST1, MST1L, MUC5B, MUC12, RFP44A, ZP3*	*ATR, BCL2L11, BMF, CBL, CRTAP, CTBP2, EHMT1, EPS1SL1, ERBIN, FBXO27, FBXW8, GNAQ, IGF1, IGF2, MAGI1, MAGI3, MOB1A, MOB1B, NEURL, VCL, VPS37C*

*p.P71S, p.P131S, p.P121S, p.P192S; ^^^p.P71L, p.P131L, p.P121L, p.P192L

### Epidermal gene expression

Only three gene transcripts were significantly upregulated, including gene paralogs *SPRR4* (FDR = 0.011, Log2FC 1.266) and *SPRR1B* (FDR = 0.026, log2FC 1.252), and four were significantly downregulated including *LAMA4* (FDR = 0.026, log2FC −1.263) and *PAX8* (FDR = 0.029, log2FC −0.785). Despite FDR > 0.05, *IFI27* (log2FC 1.565) and *WNT2* (log2FC 1.351) were noted with log2FC > 1.

### Epidermal gene signatures; gene set enrichment analysis

**Fig. 2:**
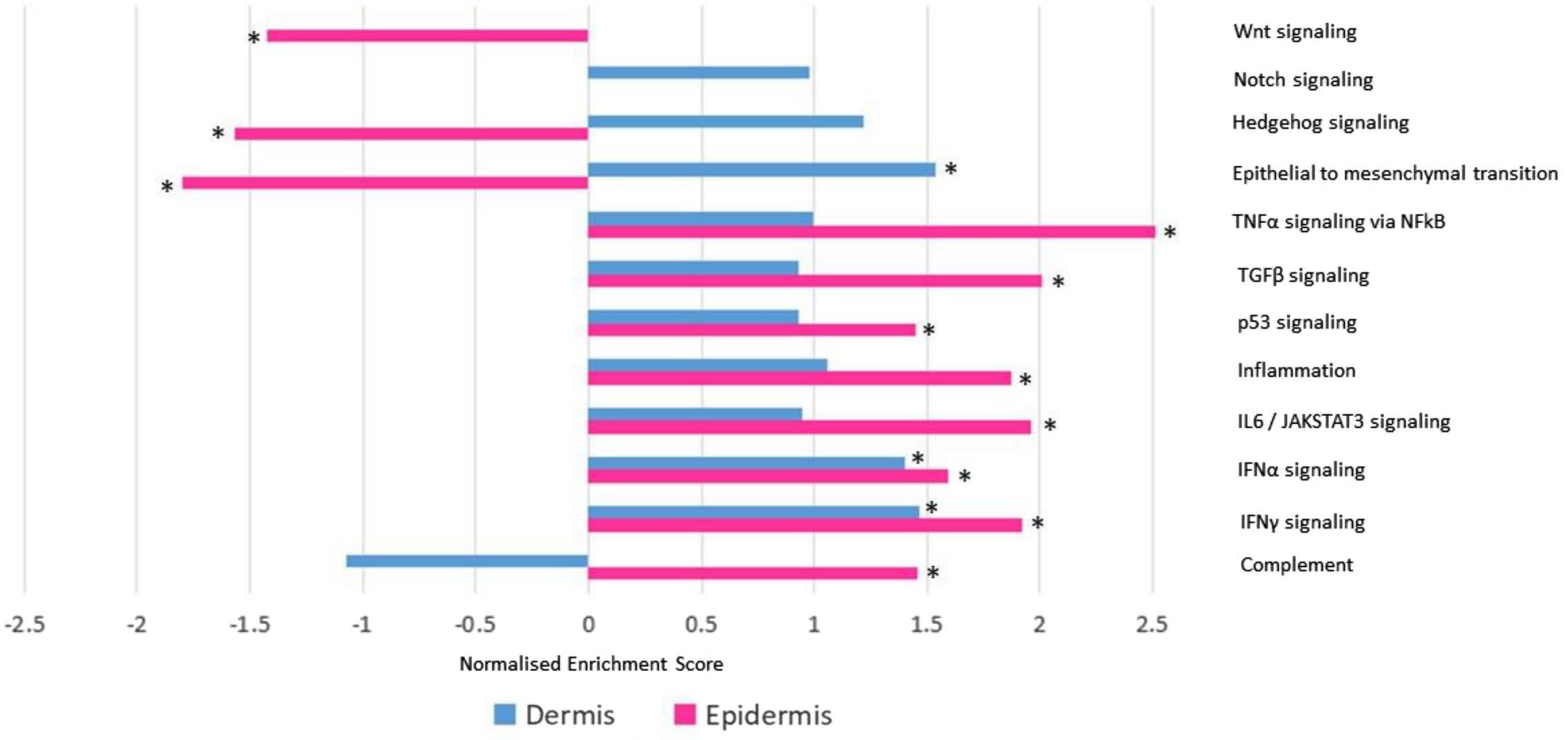
Enrichment of disease relevant Hallmark gene sets on GSEA, comparing epidermal and dermal datasets. An asterix (*) denotes significantly enriched sets (FDR < 0.25). Dermal Wnt signaling and epidermal Notch signaling were not in the top 20 differentially expressed Hallmark sets within their respective dataset and hence are not displayed graphically

Thirty-six Hallmark gene sets had significant enrichment; 16 with positive and 20 with negative enrichment. TNF-α signalling via NFkB (NES = 2.514, FDR =  < 0.001), TGF-β signalling (NES = 2.006, FDR = 0.001) and IL-6/JAKSTAT3 signalling (NES = 1.961, FDR = 0.001) were the most strongly positively enriched (Fig. 2 and Table 4).

**Table 4:** Epidermal RNA sequencing: Hallmark gene sets with significant positive or negative enrichment on GSEA, listed by NES

Hallmark gene set	NES	FDR
*Positively enriched sets*		
TNF-α signaling via NFkB	2.514	< 0.001
TGF-β signaling	2.006	0.001
IL-6/JAKSTAT3 signaling	1.961	0.001
IFNα response	1.942	0.001
Inflammatory response	1.874	0.002
Androgen response	1.821	0.002
Early estrogen response	1.800	0.003
Protein secretion	1.664	0.009
IFNγ response	1.591	0.014
Heme metabolism	1.564	0.016
KRAS signaling ↑	1.515	0.022
Complement	1.456	0.032
p53 pathway	1.451	0.031
Late estrogen response	1.438	0.032
Apoptosis	1.268	0.109
mTOR-C1 signaling	1.178	0.191
*Negatively enriched sets*		
E2F targets	-2.596	< 0.001
G2M check point	-2.375	< 0.001
Myogenesis	-1.800	0.005
Epithelial to mesenchymal transition	-1.796	0.005
MYC targets-V2	-1.754	0.006
Angiogenesis	-1.732	0.006
KRAS signaling ↓	-1.724	0.006
MYC targets-V1	-1.671	0.010
Glycolysis	-1.606	0.017
Apical surface	-1.581	0.020
DNA repair	-1.580	0.018
Hedgehog signaling	-1.571	0.018
Spermatogenesis	-1.506	0.030
Hypoxia	-1.497	0.030
Wnt-β-catenin signaling	-1.424	0.054
Mitotic spindle	-1.298	0.141
Apical junction	-1.268	0.167
Coagulation	-1.267	0.159
Oxidative phosphorylation	-1.205	0.232
Xenobiotic metabolism	-1.189	0.245

### Dermal gene expression

Ninety-three gene transcripts were significantly upregulated, 263 downregulation and 15,206 had nonsignificant differential expression (DE). A number of immunoglobulin-related genes were amongst the most strongly DEGs [(all FDR < 0.001, log2FC > 2.927). Other genes with significant positive DE included *SFRP4* (log2FC 3.277), *CXCL9* (log2FC 2.709), *COMP* (log2FC 1.664), WNT16 (log2FC 0.742), *CCL2* (log2FC 0.701), *WNT2*B (log2FC 0.576), *NOTCH4* (log2FC 0.500)]; while *MMP7* (log2FC −2.861) and *NR4A1* (log2FC −0.630) were negatively expressed.

### Dermal gene signatures; gene set enrichment analysis and PANTHER statistical enrichment analysis

**Fig. 3:**
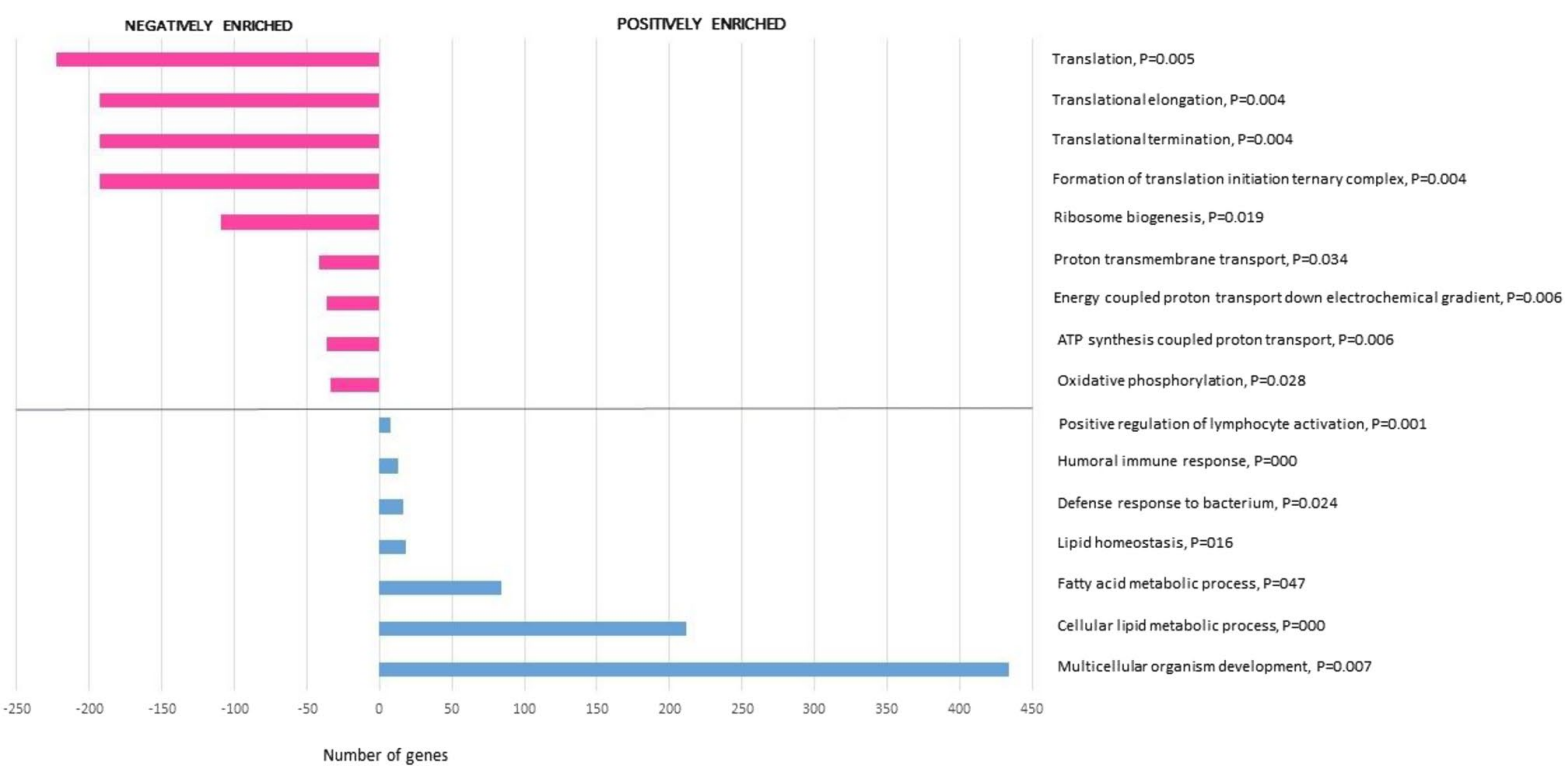
PANTHER Gene Ontology biological processes with significant positive and negative enrichment according to PANTHER enrichment test (Bonferroni correction, adjusted P-values listed next to biological process name)

Seventeen GSEA Hallmark gene sets were significantly enriched; 9 with positive and 8 with negative enrichment (Fig. 2 and Table 5). Sixteen biological processes were statistically enriched on PANTHER statistical enrichment testing; 7 with positive and 9 with negative enrichment (Fig. 3).

**Table 5:** Dermal RNA sequencing: Hallmark gene sets with significant positive or negative enrichment on GSEA, listed by NES

Hallmark gene set	NES	FDR
*Positively enriched sets*		
Bile acid metabolism	1.617	0.095
Adipogenesis	1.699	0.098
Epithelial to mesenchymal transition	1.536	0.125
Xenobiotic metabolism	1.464	0.131
Cholesterol metabolism	1.389	0.136
IFNγ response	1.402	0.145
Angiogenesis	1.422	0.147
IFNα response	1.465	0.162
Peroxisome	1.292	0.227
*Negatively enriched sets*		
Androgen response	-1.760	0.052
Oxidative phosphorylation	-1.675	0.071
Early estrogen response	-1.539	0.071
Protein secretion	-1.549	0.075
MYC targets, V1	-1.571	0.076
KRAS signaling (down)	-1.574	0.094
G2M checkpoint	-1.592	0.108
Late estrogen response	-1.468	0.113

**Fig. 4:**
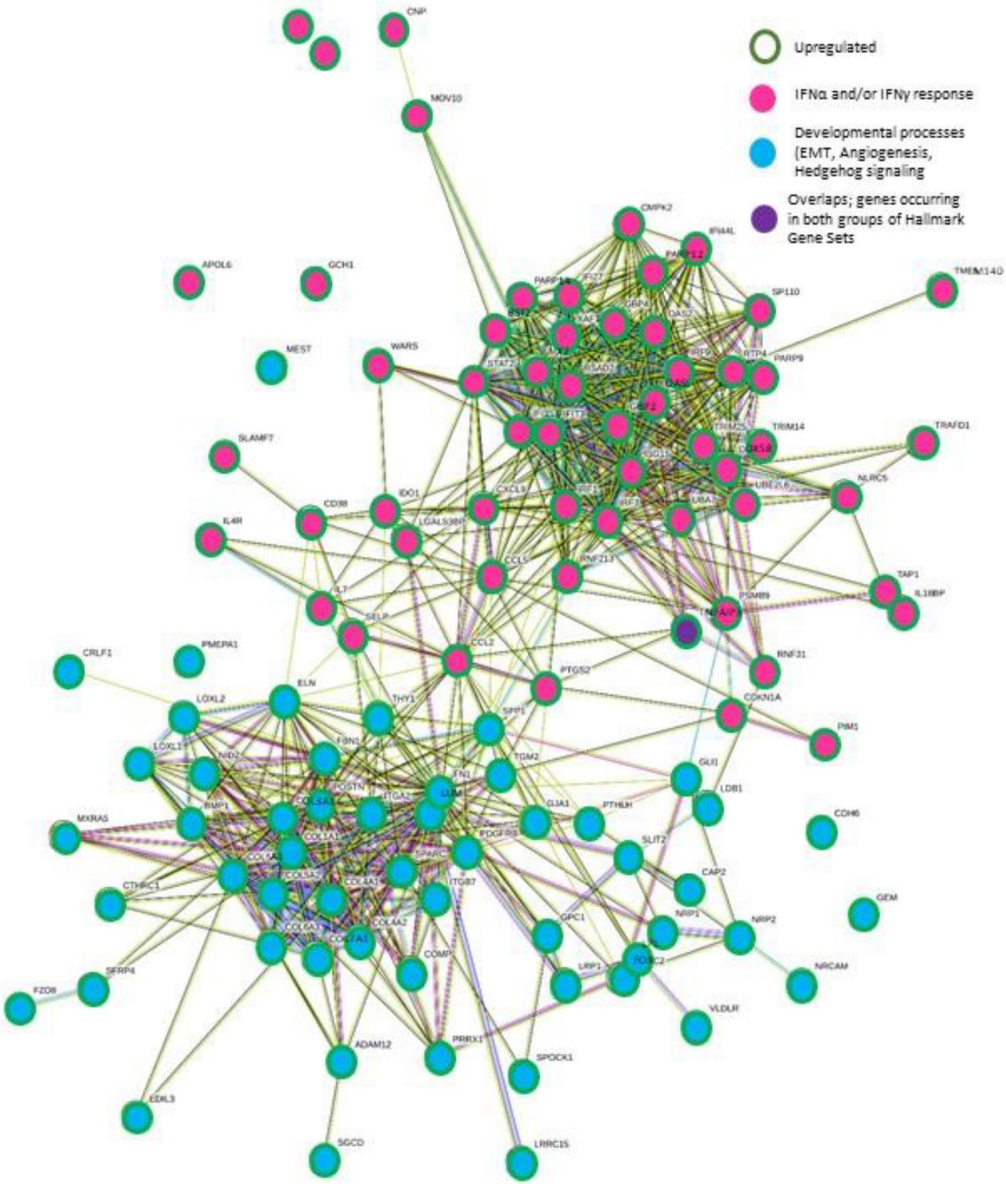
Interactions between leading edge genes within inflammatory gene sets IFN-signaling (α and γ), and developmental related gene sets of epithelial to mesenchymal transition, Angiogenesis and Hedgehog signaling, demonstrating clustering and inter-pathway interactions. Default STRING criteria used: nodes linked by evidence, with medium confidence level of 0.4

Two distinct gene expression clusters were evident from analyses; inflammatory [GSEA: IFNα response (NES = 1.465, FDR = 0.162) and IFNγ response (NES = 1.402, FDR = 0.145), and PANTHER: Humoral immune response (*P* < 0.001) and Positive regulation of lymphocyte reactivation (*P* = 0.001) see Table 6], and; profibrotic, morphogenic signatures [GSEA: Epithelial to mesenchymal transition (NES = 1.536, FDR = 0.125) and Angiogenesis (NES = 1.422, FDR = 0.147), as well as nonsignificant positive enrichment of Hedgehog signalling (NES = 1.217, FDR = 0.291), Notch signalling (NES = 0.981, FDR = 0.655) and Wnt signalling (NES = 0.453, FDR = 0.999), and PANTHER: Multicellular organism development (*P* = 0.007); 434 contributory genes including WNT (*WNT16, WNT10B, WNT2B*), hedgehog (*HHAT, HHATL*), disheveled (*DVL1, DVL2, DVL3*) and frizzled (*SMO*), HOX (*HOXA1a HOXA3, HOXA4, HOXA5, HOXA6, HOXA7, HOXA13, HOXB3, HOXB4, HOXB5, HOXB6, HOXB7, HOXC4, HOXC6, HOXC13*) and PAX (*PAX3, PAX6, PAX8*)] (Fig. 4).

**Table 6:** Dermal RNA sequencing: transcripts contributing to the three key selected positively enriched PANTHER GO-Slim Biological Processes (multicellular organism development, humoral immune response and positive regulation of lymphocyte activation) with significant upregulation

Gene symbol	Description	FDR	Log2FC	Log2CPM
*IGHG2*	Immunoglobulin heavy constant gamma 2 (G2m marker)	< 0.001	5.508	4.426
*IGHG1*	Immunoglobulin heavy constant gamma 1 (G1m marker)	< 0.001	5.162	7.118
*IGLC2*	Immunoglobulin lambda constant 2	< 0.001	4.302	4.821
*IGHG4*	Immunoglobulin heavy constant gamma 4 (G4m marker)	0.037	4.112	2.760
*IGHM*	Immunoglobulin heavy constant mu	< 0.001	4.027	5.798
*IGHA1*	Immunoglobulin heavy constant alpha 1	< 0.001	3.794	6.702
*IGLC3*	Immunoglobulin lambda constant 3 (Kern-Oz marker)	< 0.001	3.215	4.507
*IGHA2*	Immunoglobulin heavy constant alpha 2 (A2m marker)	< 0.001	2.927	4.098
*CXCL9*	C-X-C motif chemokine ligand 9	< 0.001	2.709	3.880
*SULF1*	Sulfatase 1	< 0.001	0.976	5.124
*WNT10B*	Wnt family member 10B	0.024	0.895	2.714
*WNT16*	Wnt family member 16	0.001	0.742	5.145
*COL14A1*	Collagen type XIV alpha 1 chain	0.003	0.723	7.332
*TENM4*	Teneurin transmembrane protein 4	0.032	0.668	5.754
*JCAD*	Junctional cadherin 5 associated	0.028	0.655	6.112
*NREP*	Neuronal regeneration related protein	0.017	0.613	5.547
*WNT2B*	Wnt family member 2B	0.048	0.576	5.703
*SULF2*	Sulfatase 2	0.006	0.546	7.069

**Fig. 5:**
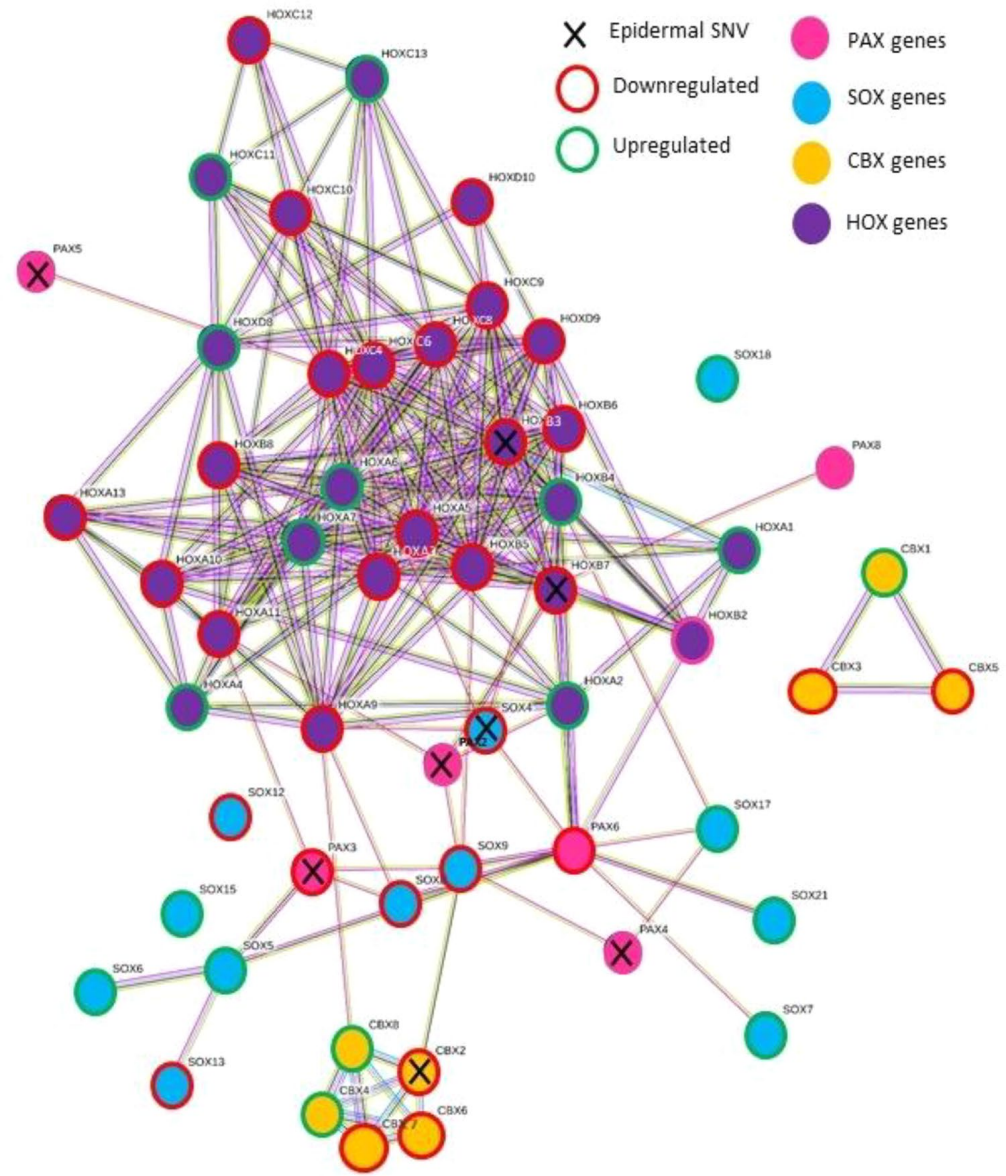
STRING network diagram demonstrating multiple strong and overlapping interactions between PAX, HOX, SOX and CBX genes with protein or non-protein coding epidermal SNVs on WGS and/or differential epidermal or dermal expression on RNA-seq. Nodes linked by evidence with medium confidence level of 0.4 (default STRING criteria)

Many HOX, PAX, SOX and CBX genes were impacted across all three epidermal/dermal and genomic/transcriptomic datasets (Fig. 5).

**Fig. 6:**
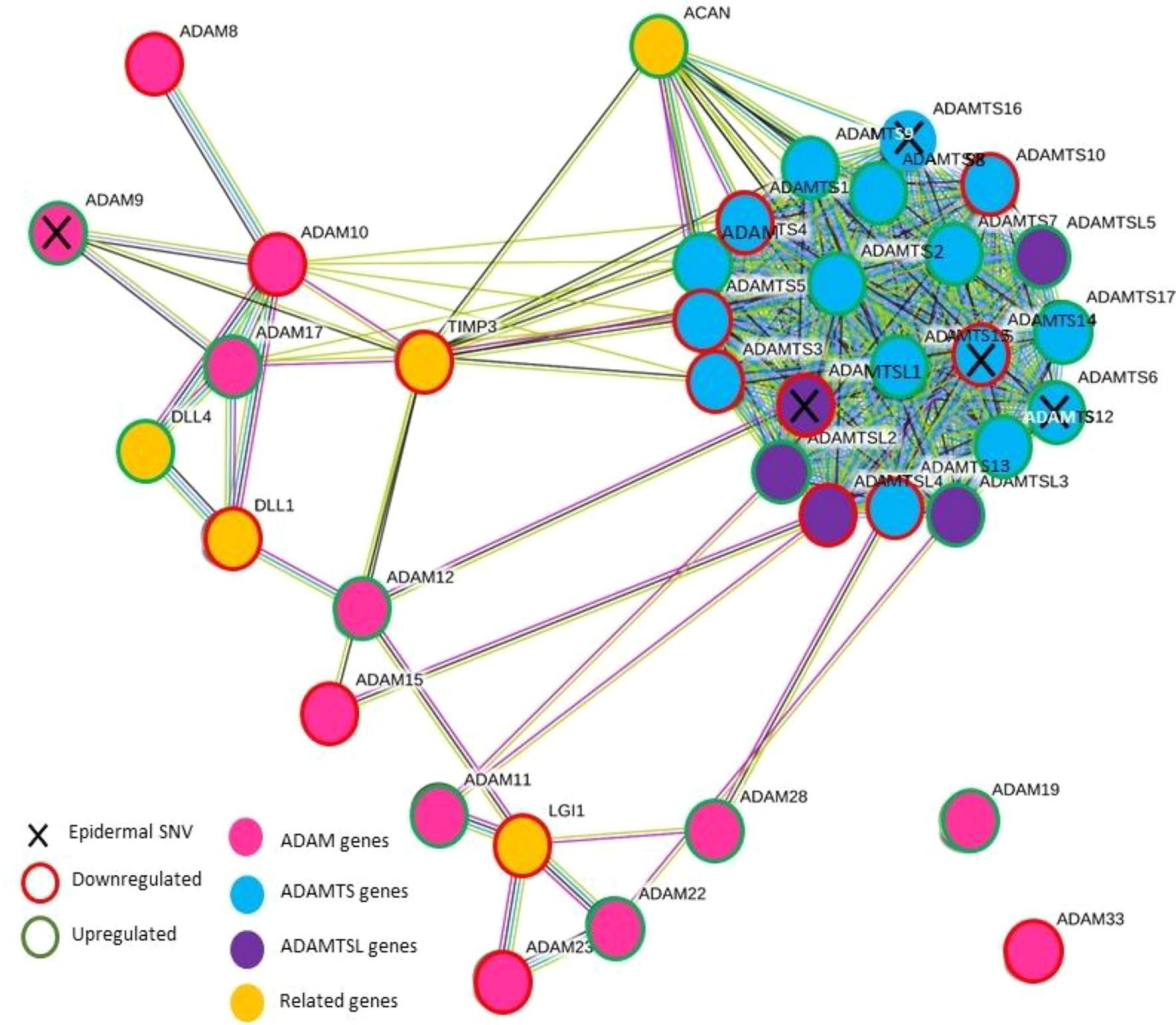
STRING network diagram of all ADAM, ADAMTS and ADAMTSL proteases with epidermal SNVs and/or epidermal and/or dermal differential RNA expression. Nodes linked by evidence, with medium confidence level of 0.4 (default STRING criteria). Further genes with strong links to the ADAM, ADAMTS and/or ADAMTSL proteins were also included (via STRING extended analysis); two of which were the ‘delta like canonical notch ligands’ (1 and 4); linking the ADAM, ADAMTS and ADAMTSL proteins, to notch signalin

Thirty-two members of the ADAM, ADAMTS and ADAMTSL super-family were nonsignificantly DE in the dermis (13 downregulated and 19 upregulated) and 12 in the epidermis (6 upregulated and 6 downregulated). Overall, 50 ADAM/ADAMTS-family genes were affected across all three datasets, including the potentially highly pathogenic (according to criteria described in Fig. 1) nonsynonymous SNVs in *ADAMTS16* and *ADAMSTL1* (Fig. 6).

### Candidate genes and pathways based on epidermal genomic and epidermal and dermal transcriptomic profiles

Based on the WGS and RNA-seq results, a number of gene candidates were selected; some for further study. Selected epidermal candidate genes included *ADAMTS16, ADAMTSL1* and the inflammatory and profibrotic *TGF-β1* and *JUNB*. Selected dermal candidates included members of some developmental and morphogenic signaling pathways; *SFRP4, SIX1, WNT2* and *NOTCH4*. Key characteristics of these genes and justification for their selection as candidates are detailed in Table 7.

**Table 7:** Descriptive and statistical characteristics of selected gene candidates in epidermal and dermal tissue

Gene symbol	Description	FDR	Log2FC	Log2CPM	Notes/data related Justification
*Epidermal candidates*					
*ADAMTS16*	ADAM Metallopeptidase With Thrombospondin Type 1 Motif 16	N/A	N/A	N/A	WGS data: Novel variant Denoted deleterious by PolyPhen2,PROVENA and SIFT scores. CADD score 33 Only variants graded as High and subcategorised as Level 1 for disease relevance and pathogenicity Known links to fibrosis
*ADAMTSL1*	ADAMTS Like 1	N/A	N/A	N/A	WGS data only: Novel variant Denoted deleterious by PolyPhen2,PROVENA and SIFT scores. CADD score 30 Only variants graded as High and subcategorised as Level 1 for disease relevance and pathogenicity Known links to fibrosis
*LAMA4*	Laminin subunit alpha 4	0.026	− 1.26	2.21	RNA-seq data: Significant FDR, log2FC < -1 Known links to fibrosis in other organs Plausible involvement in epidermal-dermal interactions in pathogenic mechanisms
*IFI27*	Interferon Alpha Inducible Protein 27	0.952	1.565	5.721	Only epidermal transcript with log2FC > 1.5 Epidermal GSEA, Hallmark gene set leading edge gene: IFNα signaling (NES = 1.924, FDR = 0.0011) IFNγ signaling (NES = 1.591, FDR = 0.014) Plausible epidermal early ‘damage’ signal, with links to downregulation of NR4A1
*TGF-β1*	Transforming Growth Factor Beta 1	0.990	-0.036	5.362	Key initiator and mediator of fibrosis Epidermal expression never specifically investigated in morphoea Overall signaling (TGF-β signaling Hallmark set) strongly positively enriched via GSEA analysis (NES = 2.006, FDR = 0.001)
*JUNB*	JunB Proto-Oncogene, AP-1 Transcription Factor Subunit	0.952	0.424	7.939	Relatively high log2CPM of 7.939 Epidermal GSEA, Hallmark gene set leading edge gene in TGF-β signaling Hallmark set (NES = 2.006, FDR = 0.001)
*PAX3*	Paired box gene 3	N/A	N/A	N/A	Epidermal WGS: nonsynonymous protein coding deleterious SNV Links to epidermal upregulation of PAX8 as well as many other PAX, HOX, SOX and CBX genes in both epidermal and dermal datasets; many with links to fibrosis and SSc
*Dermal candidates*					
*SFRP4*	Secreted Frizzled Related Protein 4	< 0.001	3.277	5.582	Frizzled related protein with significant differential expression and log2FC > 3 Dermal GSEA, Hallmark gene set leading edge gene: Epithelial to mesenchymal transition (NES = 1.536, FDR = 0.125), highest ranked leading edge gene
*SIX1*	SIX Homeobox 1	0.641	2.333	2.529	Homeobox gene with the highest log2FC
*WNT2*	Wnt Family Member 2	0.061	1.793	2.283	Only Wnt signaling with log2FC > 1.5 Differential expression approaching significance Dermal GSEA, Hallmark gene set leading edge gene: Notch signaling, top 20 positively enriched sets (NES = 0.980, FDR = 0.655), highest ranked leading edge gene PANTHER statistical enrichment test: Present within the significantly enriched Multicellular organism development gene set (PANTHER GO-Slim Biological Process), *P* = 0.007
*NOTCH4*	Notch Receptor 4	0.008	0.500	5.631	Only significantly differentially expressed NOTCH gene Relatively high log2CPM
*NR4A1*	Nuclear Receptor Subfamily 4 Group A Member 1	0.003	−0.63	4.81	Significant dermal downregulation Downregulated by IFI27 (see above) Endogenous regulator of TGF-β1 signaling and known involvement in fibrotic processes
*CXCL9*	C-X-C Motif Chemokine Ligand 9	< 0.001	2.71	3.88	Inflammatory IFN response related gene with significant and strong differential expression Dermal (and epidermal) GSEA, Hallmark gene set leading edge gene: Contribution to the leading edge gene profile for IFNγ signaling in both the dermis and epidermis Suggested as a biomarker in morphoea
*CCL2*	C-C Motif Chemokine Ligand 2	0.034	0.7	4.34	Inflammatory IFN response related gene with significant differential expression Dermal (and epidermal) GSEA, Hallmark gene set leading edge gene: Contribution to the leading edge gene profile for IFNγ signaling in both the dermis and epidermis Over-expressed amongst morphoea patients included in the Milano et al. ‘intrinsic gene subset’ scleroderma study and has been isolated to dermal macrophages in morphoea

### RT-qPCR and immunohistochemistry validation of selected epidermal and dermal gene candidates

Two key candidate genes were validated by RT-pPCR in this study; TGF-β1 and JUNB. These were from the strongly over-expressed and highly disease-relevant TGF-β signaling gene set. *TGF-β1* is the recognised orchestrator of fibrosis and the role of its epidermal production and expression have not been specifically investigated in morphoea. *JUNB* is also a key player in TGF-β signaling and hence with its relatively high log2CPM, *JUNB* was selected as the second validation candidate, keeping both genes for qPCR from the TGF-β signaling gene set (NES = 2.006, FDR = 0.001).

**Fig. 7:**
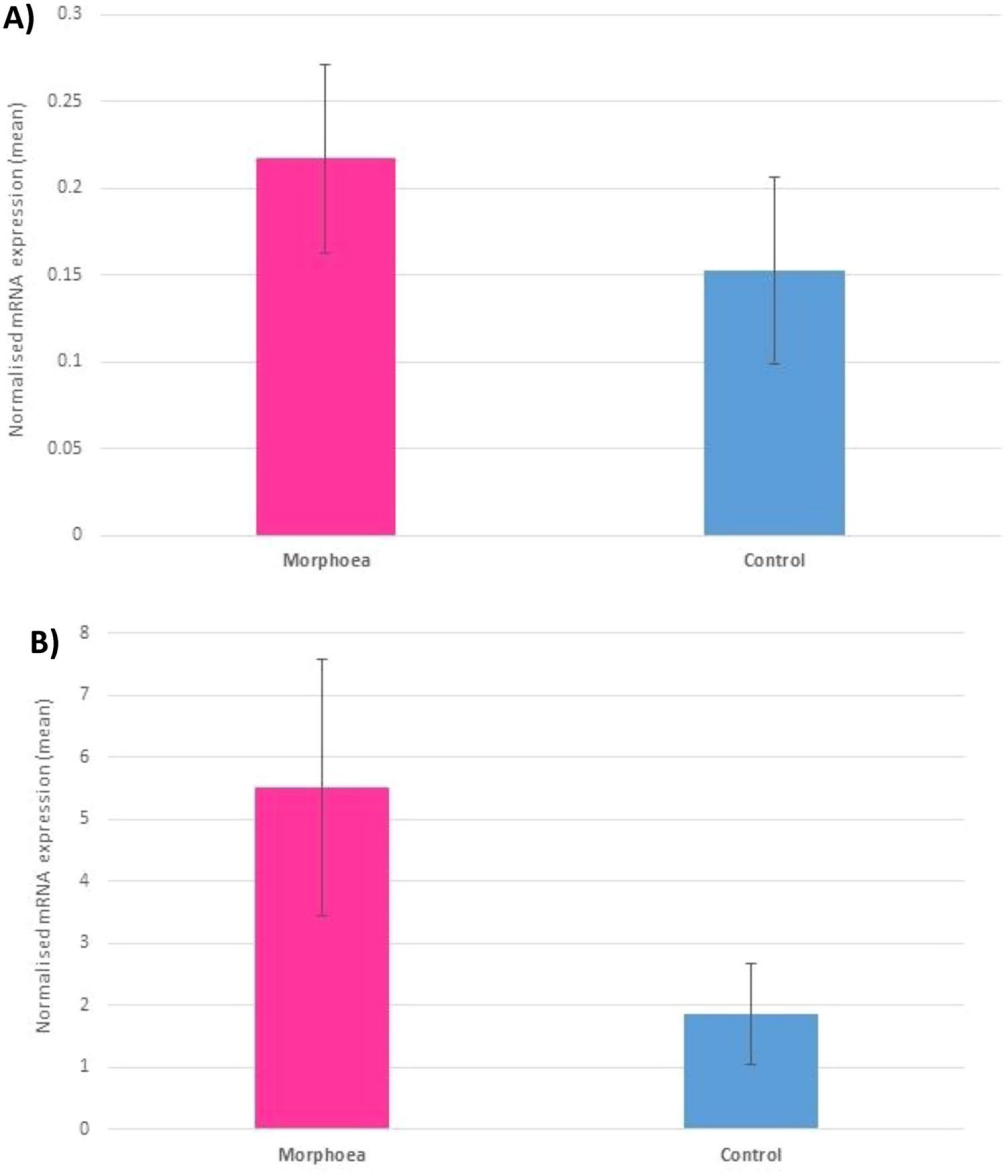
RT-qPCR validation for key epidermal upregulated TGF-β signaling genes, mean expression levels as normalised copy number; **A** TGF-β1, **B** JUNB

Expression of TGF-β1 and JUNB was higher in morphoea affected epidermis compared to the contralateral site-matched unaffected epidermis in all samples, but this trend was not significant (TGF-β1; *P* = 0.476, JUNB; *P* = 0.105, Fig. 7).

*WNT2* was selected for validation via IHC on formalin-fixed, wax-embedded paraffin whole skin sections. *WNT2* was highlighted by dermal transcriptomic profiling, subsequent pathway analysis and is a member of the developmental morphogenic pathways which are of particular relevance to the anatomical patterning in morphoea and its pathogenesis. Of note, *WNT2* was also highlighted by epidermal RNA-seq.

In the dermis, *WNT2* was the only Wnt signaling gene with log2FC > 1.5 (log2FC = 1.79), its FDR approached significance (FDR = 0.061), it was a leading edge gene (highest ranked) within the positively enriched Notch signaling Hallmark gene set within dermal GSEA data and was also present within the significantly enriched Multicellular organism development gene set (PANTHER GO-Slim Biological Process; *P* = 0.007).

**Fig. 8:**
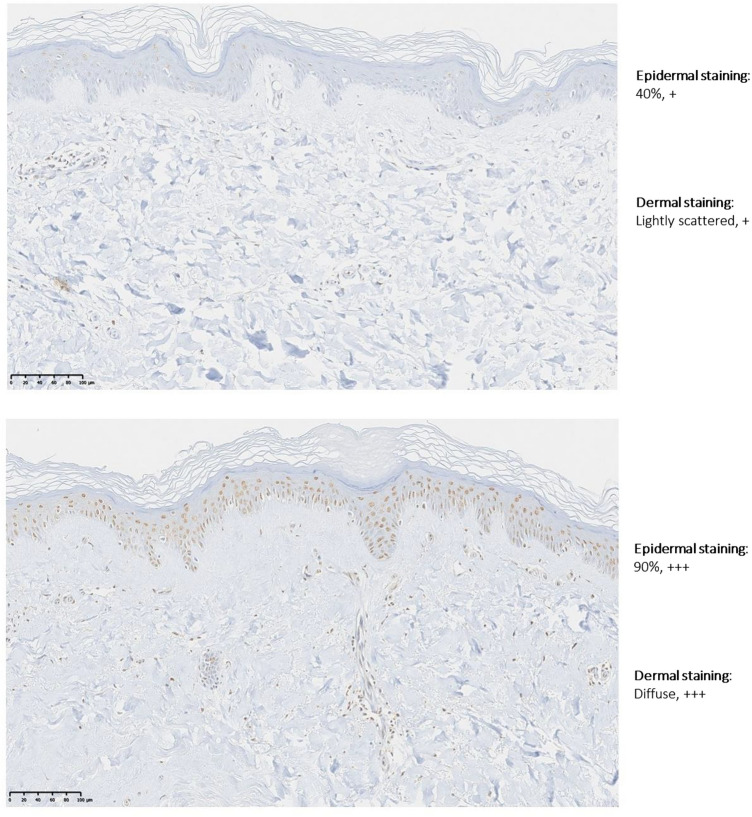
High power images of immunohistochemical staining with WNT2 antibody; unaffected control skin (above) and morphoea affected contralateral site-matched skin (below); study participant 15

*WNT2* staining demonstrated discernible staining differences between morphpea-affected and unaffected control skin in both epidermis (4 of 5) and dermis (3 of 5) (Fig. 8).

## Discussion

In this study, WGS did not identify a single common somatic mutation occurring in all four epidermal samples taken from LM-affected skin, or a commonly affected gene across all study samples. To our knowledge, this is the first study to investigate the presence of primary genomic variation in morphoea skin. This critical finding provides robust evidence against primary genomic epidermal segmental mosaicism-related aetiology in adult-onset LM. There are several clinical complexities of LM supporting more multifaceted aetiopathogenesis. LM may not be truly Blaschkoid [8], morphoea is a dermal pathology, has vast clinical heterogeneity with complex patterning and morphology [4, 30] and is not congenital.

Accordingly, we identified 861 epidermal SNVs, including 119 protein-coding variants, many with medium to high disease relevance and potential pathogenicity, providing possible support for complex polygenic epidermal mosaicism in LM [31, 32].

The ADAM/ADAMTS-family genes were widely affected across all three datasets, including potentially highly pathogenic nonsynonymous SNVs in *ADAMTS16* and *ADAMSTL1*, possibly pointing to their pathogenic role in morphoea. These proteins/proteases are ECM-regulators implicated in embryological morphogenesis, skin development, wound healing, fibrosis [33–36], rare primary fibrotic genetic disorders [37, 38], SSc and keiloidal morphoea [39, 40]. Using site-matched tissue-pair methodology, Badshah et.al. recently demonstrated upregulated *ADAMTS8* in LM fibroblasts and whole-skin, hypothesising *ADAMTS8*’s role in tissue atrophy [41]. Whilst links between the ADAMTS/ADAMTSL’s and their precise functions in morphoea are unclear, their possible role in LM is further supported by our findings.

**Fig. 9:**
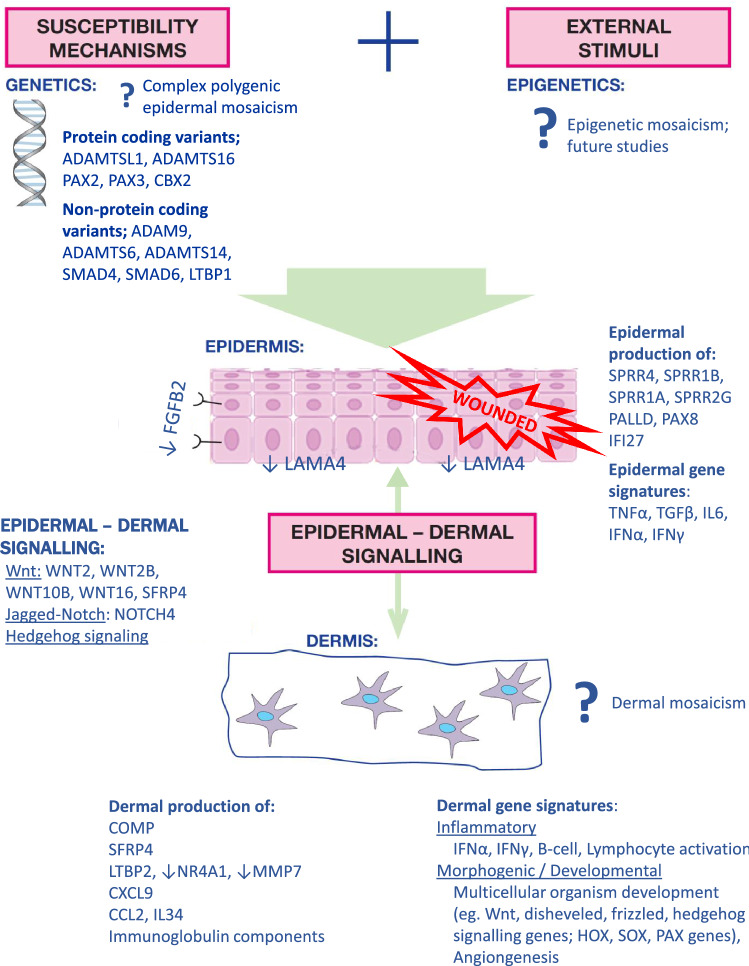
Multicomponent morphea etiopathogenesis; summary of key epidermal and dermal genes involved in morphea, as highlighted by NGS of paired epidermal and dermal tissue samples in this study

Corroborating the potential key role of the epidermis in morphoea pathogenesis, we demonstrated a structurally active, proliferative and differentiating epidermis, with significant overexpression of *SPPRs, PALLD, WNT2*, other cell cycle/cell division (such as p53 and KRAS signalling) and apoptosis-related gene pathways, along with significant down-regulation of checkpoint and DNA repair-related genes (such as G2M DNA checkpoint and E2F targets) (Fig. 9).

We also demonstrated an inflammatory and profibrotic epidermal gene signature, which corresponds to the early inflammatory and profibrotic disease phases previously mapped by blood cytokine profiles [42–46]. A Th1 response (IL-2, TNF-α and IL-6) seen in the first year, is followed by a Th17 response (*IL-1, IL-17, IL-22* and *TGF-β*) and Th2 cytokines (*IL-4* and *IL-13*) [47]. Accordingly, the three Hallmark gene sets with the strongest significant positive enrichment in this study were TNF-α signalling via NFkB, TGF-β signalling and IL-6/JAKSTAT3 signalling; all suggesting early active inflammatory and fibrotic phase disease (Fig. 9). This was despite study samples being from LM of at least 3-years duration and not all demonstrating an inflammatory clinical phenotype; supporting an ongoing disease-driving role of the epidermis.

Importantly, in recently published work evaluating transcriptomic whole-skin profiles of pediatric-onset morphoea, healthy controls, active and inactive disease were compared, and JAK/STATs were highlighted as the most prevalent DE pathway [48]. By separating the epidermis and dermis, we have highlighted that this signature may originate from the epidermis, promoting ongoing dermal disease activity. These findings provide further support for future studies to better elucidate precise pathogenic JAK/STAT-related mechanisms in morphoea and the use of therapeutic JAK-inhibitors in sclerotic skin disease [49].

Finally, the epidermal molecular picture was also that of a ‘wounded epidermis’, similar to the epidermal phenotype demonstrated in SSc [10, 50, 51]. *TGF-β* is a key orchestrator of wound healing responses, also propagating pathological fibrosis [52]. Isolating a strongly enriched *TGF-β* signature in morphoea epidermis is unique, significant, and could provide impetus for further study of local *TGF-β* inhibition in appropriate clinical scenarios of superficial disease (e.g. with pirfenidone) [53]. However, precisely whether these signals are originating in the epidermis, or due to secondary unchecked positive feedback from the dermis, remains unclear.

Relevantly, epidermal *IFI27* was upregulated (nonsignificant, but with the dataset’s highest log2FC). It is known to induce IFNγ-related epidermal apoptosis. We saw significant upregulation of the epidermal Apoptosis gene set, and epidermal and dermal IFNα and IFNγ responses. IFN-signalling has been widely implicated in SSc and morphoea [11, 48, 54]. IFNγ-related chemokines and their receptors may stimulate fibroblasts, including in morphoea [46, 48, 55]. *CXCL9* was significantly upregulated in morphoea dermis in our study, and it has previously been suggested as a disease biomarker [46, 55].

Importantly, *IFI27* negatively regulates *NR4A1* [54], which was significantly downregulated in the dermal dataset. In turn, *NR4A1* is an endogenous *TGF-β* inhibitor [56]. Fibrotic diseases appear to utilise this *NR4A1*-dependent mechanism to enable persistent *TGF-β* signaling and deregulated fibrosis and *NR4A1* agonists inhibit laboratory-induced fibrosis of the skin, lung, liver, and kidney in mice [56, 57].

Clues to another potential inciting epidermal ‘damage’ signal in morphoea lie in the significant downregulation of *LAMA4.* Laminins are extracellular matrix (ECM) glycoproteins involved in differentiation, cell adhesion, signaling, migration, and form a key non-collagen component of the dermo-epidermal junction (DEJ) [54]. Related DEJ disruption could plausibly enhance epidermal-dermal communication and/or act as an initiating ‘damage’ signal, inciting proinflammatory and profibrotic dermal responses. Correspondingly, *LAMA4*-deficiency has been linked to cardiac [58–60] and renal fibrosis [61].

Individual dermal-genes demonstrated far greater DGE compared to the epidermis, suggesting dermal factors are more disease-specific in morphoea; in keeping with its predominantly dermal pathology. Two distinct DGE clusters were identified; inflammatory and profibrotic. The inflammatory signature, with significant upregulation of Humoral immunity, Lymphocyte activation and IFN-response-related genes, validates and adds to the limited morphoea gene expression data currently available [11, 48, 62]. This corroborated over-expression of IFN-signalling has an immediate foreseeable opportunity for potential therapeutic exploitation via anifrolimab, FDA-approved for systemic lupus erythematosus. Interestingly, KRAS-signalling has been identified as a potential biomarker for disease activity [48]. We demonstrated significant downregulation of inhibitory KRAS-signalling in the dermis and upregulated KRAS-signalling in the epidermis also. All our cases had disease activity as demonstrated by LoScAT-activity scores of greater than zero (progressive or stable disease activity) (Tables 1, 4 and 5).

In the profibrotic DGE cluster, upregulated genes involved in embryogenesis and oncogenesis was seen such as Wnt, Hedgehog, dishevelled, frizzled family, HOX and PAX. PAX and HOX genes were specifically highlighted by PANTHER pathway analysis of dermal RNA-seq data. These families of biologically and functionally related developmental genes were collectively impacted in all three data sets (epidermal WGS, epidermal RNA-seq and dermal RNA-seq). HOX genes are the key orchestrating genes involved in fibroblast PI [12, 13, 63–65]. Related location-specific gene signatures confer developmental patterning, position and help determine downstream differentiation of site-specific mesenchymal cells [13, 66]. The genetic origin of fibroblasts can also alter their crosstalk with overlying keratinocytes [67]. Several HOX genes have shown significant DE in affected SSc-skin compared to unaffected skin [68] and related SOX genes have also been implicated in fibrosis and SSc [23, 69]. Accordingly, one can deduce the feasible role HOX and related developmental and patterning genes could play in morphoea aetiopathogenesis and observed clinical patterning of non-linear subtypes. Indeed, their involvement in ‘dermal mosaicism’ has been suggested.

It is also suggested that via its regulation of dermal development, epidermal Wnt- signalling could account for the Blaschkoid distribution of dermal dermatoses, including Focal Dermal Hypoplasia [70]. Twelve Wnt-signalling genes contributed to the upregulation of the GO-Slim Biological Process of Multicellular organism development; *WNT2B, WNT10B* and *WNT16* with significant DE. WNT2 was significantly upregulated in the epidermis, approached significance in the dermis (FDR = 0.061) and both these RNA-seq results were validated with IHC whole skin staining. Correspondingly, *WNT2, WNT3A* and *β-catenin* have previously demonstrated increased activity via IHC staining in both SSc and morphoea [71] and the role of Wnt-signalling in morphoea is established [20, 55, 71–75]. Dermal *SFRP4* was also significantly upregulated and recent data demonstrated the upregulation of *SFRP2* in morphoea dermal fibroblasts [55]. SFRPs are homologous to the Wnt-binding site on frizzled proteins and, therefore, modulate Wnt-signalling via direct interactions [54]. Interestingly, *SFRP4* expression in the myocardium is associated with an apoptotic-related gene expression profile [54], feasibly associating its overexpression in morphoea to a disease-related damage signal.

Limitations of this study include its cross-sectional nature, small datasets and limited validation of transcriptomic data. It is also impossible to differentiate primary from secondary gene expression changes or to adjust for treatment effect.

In summary, despite the often assumed Blaschkoid distribution of LM, data from this study indicate the absence of a single epidermal developmental somatic mutation responsible for disease causation. Instead, this study’s molecular (genomic and transcriptomic) and tissue (epidermis and dermis) layered approach highlights possible polygenic epidermal mosaicism in initiating a complex multicomponent disease aetiopathogenesis. A wounded epidermal phenotype could, perhaps via Wnt-signalling, depletion of NR4A1 and other complex tissue layer crosstalk, contribute to the consequent inflammatory dermal fibrosis of morphoea, with its variable patterning possibly explained, at least in part, by the involvement of HOX, SOX, PAX and WNT developmental patterning genes (Fig. 9).

## Supplementary Information

Below is the link to the electronic supplementary material.

Supplementary file1 (DOCX 46 KB)

## Data Availability

The data that support the findings of this study are available upon reasonable request to the corresponding author, after validation by co-authors.

## Abbreviations

BGI: Beijing Genomics Institute
C: Control skin (denoting a skin sample taken from a site unaffected by morphoea; contralateral site-matched pair)
CADD: Combined annotation-dependent depletion
CCL: CC chemokine ligand
CNS: Central nervous system
COMP: Cartilage oligomeric matrix protein
CPM: Counts per million
CTGF: Connective tissue growth factor
CXCL: Chemokine C-X-C (motif) ligand
DE: Differentially expressed
DGE: Differential gene expression
DNA: Deoxyribonucleic acid
ECM: Extracellular matrix/extracutaneous manifestations
EMT: Epithelial to mesenchymal transition
ES: Enrichment score
ET-1: Endothelin 1
ExAC: Exonerated aggregation consortium
FC: Fold change
FDR: False discovery rate
FGF: Fibroblast growth factor
Fli1: Friend leukaemia virus integration 1
GSEA: Gene set enrichment analysis
H&E: Haematoxylin and eosin
IFN: Interferon
IHC: Immunohistochemistry
IL: Interleukin
LoSCAT: Localised scleroderma cutaneous assessment tool
LM: Linear morphoea
LTBP: Latent transforming growth factor beta binding protein
M: Morphoea-affected skin (denoting a sample from skin affected by morphoea)
MAC: Morphoea in adults and children cohort
MAF: Minor allele frequency
mLoSDI: Modified localised scleroderma damage index
mLoSSI: Modified localised scleroderma severity index
MMF: Mycophenolate mofetil
MMP: Matrix metalloproteinase
mRSS: Modified Rodnan skin score
MTX: Methotrexate
NGS: Next-generation sequencing
NES: Normalised enrichment score
NFkB: Nuclear factor kappa-light-chain-enhancer of activated B cells
ng: Nanogram
NLRP3: NACHT, LRR and PYD domains-containing protein 3
PANTHER: Protein analysis through evolutionary relationships
PCR: Polymerase chain reaction
PDGF: Platelet-derived growth factor
PI: Positional identity
PPAR-γ: Peroxisome proliferator-activated receptor gamma
PROVEAN: Protein variation effect analyser
PUVA: Psoralen ultraviolet-A
QC: Quality control
qPCR: Quantitative polymerase chain reaction
QoL: Quality of life
RNA: Ribonucleic acid
RNA seq: RNA sequencing
RT-qPCR: Reverse transcriptase quantitative polymerase chain reaction
SIFT: Sorting intolerant from tolerant
SNP: Single nucleotide polymorphism
SNV: Single nucleotide variant
SSc: Systemic sclerosis
TGF-β: Transforming growth factor beta
TIMP: Tissue inhibitor of metalloproteinase
TNF-α: Tumour necrosis factor alpha
µL: Microlitre
WES: Whole exome sequencing
WGS: Whole genome sequencing
