# Supplementary material for: Unravelling morphoea aetiopathogenesis by next-generation sequencing of paired skin biopsies
Source: Arch Dermatol Res. 2023 Mar 13;315(7):2035–56. doi: 10.1007/s00403-023-02541-5 (PMC10366313; doi:10.1007/s00403-023-02541-5)
Supplement: Supplementary file 1 — Supplementary file1 (DOCX 46 KB) [file 403_2023_2541_MOESM1_ESM.docx]

**Supplemental Methods**

***Sample selection for each dataset***

Sample selection considerations for each dataset (epidermal DNA for WGS, epidermal RNA for RNA-seq and dermal RNA for RNA-seq) included clinical homogeneity between samples, disease activity and morphology, as well as DNA and RNA quality and quantity. Preferred samples for RNA-seq were those from the same study participants that underwent epidermal WGS, to enable direct comparison and integrational molecular and tissue analysis of the data. However, three of the matching epidermal RNA isolates failed quality control due to low RNA quantity. Accordingly, alternative tissue pairs for epidermal RNA-seq were selected, to maintain the strength of utilising intra-patient contralateral pair methodology, but with ability to directly compare to epidermal WGS data (and subsequent dermal RNA-seq data) forgone (Table 1).

***DNA isolation, whole genome sequencing and analysis; epidermis***

DNA was isolated from paired epidermal tissue utilising the DNeasy micro Kit (QIAGEN, GmbH Hilden Germany) protocol, but with a repeated elution step in a further 200µl of Buffer AE to maximise DNA yield. Quality control via NanoDrop ND-8000 spectrophotometer (Thermo-Scientific, Wilmington, Delaware, USA) and RT-qPCR was performed.

Isolated DNA from four epidermal tissue pairs underwent WGS (Beijing Genomics Institute (BGI); low input protocol HiSeq X Ten System (Illumina, San Diego, CA, USA) to 60-70× coverage. Sequence reads were aligned to the GRCh38 reference genome using bwa-mem(1). Sambamba marked duplicate reads(2), and somatic variant calling was performed using MuTect2 as a part of Genome Analysis Toolkit (GATK) v.3.8 and v.4Beta(3-5) according to best practices workflow. Variants were annotated with ANNOVAR(6).

***Network analyses***

Four analyses were completed (STRING online database (v11), for; detailed in Supplemental methods section.

• each paired-sample

• genes or SNVs shared across each dataset

• all datasets pooled together

• manual scrutinisation of all protein coding SNVs for potential disease relevance.

***Epidermal single nucleotide variant classification***

Further consideration and classification of genomic variants thus far highlighted by network analyses was performed next. Consideration was now given to the highly important variant characteristics of population allele frequency (MAF) based on the ExAC database (non-Finnish European) and predicted pathogenicity (based on four predictive algorithms; PolyPhen-2, PROVEAN, SIFT and CADD score), as well as a quantitate approach to disease relevance. Pathogenicity is the likelihood that an SNV in the human genome will have a deleterious impact. Algorithms have been devised to predict pathogenicity and commonly used datasets include PolyPhen-2, Protein Variation Effect Analyser (PROVEAN), Combined Annotation-Dependent Depletion (CADD) and Sorting Intolerant From Tolerant (SIFT)(7-11). Each of the different algorithms used in these prediction tools have variable strengths and weaknesses. The programs predict function based on a set of known mutations which is different for each. In addition, different algorithms are used, based on amino acid conservation and molecular interactions of the tertiary structure. Hence the deleteriousness of a particular SNV can be differentially predicted by each program and the concurrent use of multiple prediction tools is therefore generally recommended, and used in this paper. Each uses a different scoring system.

Accordingly, a two stage gene variant classification system was created and implemented (Figure 1).

Variant disease relevance; all genes identified in the previous section, as well as all additional nonsynonymous protein coding variants, were graded as Very High, High or Medium with regards to functional relevance to morphoea and its aetiopathogenesis. This was based on what is known regarding the gene and its function, its involvement in disease relevant pathways and any known involvement in related sclerotic and / or inflammatory pathologies.

*Definitions of each disease relevance grade were:*

Very high *(known pathogenic involvement)*

Variants occurring in genes which are known to be specifically involved in the pathogenesis of morphoea or related disease entities such as SSc.

High *(links to known pathogenic mechanisms)*

Variants occurring in genes which are directly linked by function to genes / proteins known to be involved in the pathogenesis of morphoea or related disease entities such as SSc.

Medium *(theoretical relevance)*

Variants occurring in genes where involvement in morphoea pathogenesis makes biological sense based on what is known regarding gene function, however with no published direct pathological links to morphoea or related disease entities such as SSc.

Variants occurring in two or more study participants which have no apparent disease relevance were graded as ‘medium’ due to their increased possible unrecognised disease relevance suggested by co-occurrence in multiple data sets.

*Variant pathogenicity and allele frequency;*

Criteria were then applied to further classify these disease relevant and graded variants, by assigning them to Level 1, Level 2, Level 3 and Level 4. Levels were based on differentially stringent cut-offs for allele frequency (<1.0% or ≥1.0%), and the likelihood of deleterious impact of a variant, as assessed by the Polyphen-2, PROVEAN, SIFT and CADD score (>15 was defined as deleterious, ≤15 innocuous) algorithms.

Accordingly, protein coding nonsynonymous variants graded as high for disease relevance and classified as Level 1 for pathogenicity are of highest interest as potential gene candidates for further study. Those graded as low for disease relevance and classified as Level 4 for pathogenicity are of lowest interest as potential candidates. Variants without an assignable level (such as synonymous variants selected from initial network analysis due to their possible disease relevance) were graded only and noted separately.

***RNA isolation, sequencing and analysis; epidermis and dermis***

Total RNA was isolated from paired epidermal and dermal tissue utilising the RNeasy mini-kit (Qiagen, Crawley, UK) according to the manufacturer’s instructions. Total RNA concentration, quality and purity was determined by NanoDrop ND-8000 spectrophotometer (Thermo-Scientific, Wilmington, Delaware, USA) and RT-qPCR.

Selected samples underwent RNA-seq (BGISEQ-500, 20million-reads-per-sample). Read alignment to human genome reference build 38 (GRCh38) with TopHat v2.1.1 was completed, producing BAM files(12). These were checked for quality utilising RSeQC(13). SAMtools was used for quality filtering of reads. FeatureCounts was utilised to count reads and these were input into differential gene expression analysis(14). R version 3.3.2 and the R package edgeR were utilised for gene expression analysis and statistical significance calculated using the generalised linear model likelihood ratio test. Differential expression was calculated as fold-change in log2 scale (15). Genes were considered to have significant DE with a p-value of <0.05 after Benjamini-Hochberg adjustment controlling for false discovery rate.

***Gene set enrichment and pathway analyses***

Further analysis of differentially expressed genes was undertaken via key pathway and network analysis tools. Gene set enrichment analysis (GSEA) was employed for both epidermal and dermal RNA-seq datasets. the Molecular Signature Database (MSigDB) Hallmark was used, which includes 50 GSEA gene sets(16). An enrichment score (ES) was calculated for each GSEA set. This was then normalised (NES) and used to determine if the collective set of genes, specifically the leading edge genes, were differentially expressed between morphoea affected and site-matched unaffected control skin (positive NES = upregulated, negative NES = downregulated). As per standard protocols, enrichment was reported as significant if FDR was less than 0.25(17). GSEA profiles, including enrichment plots, leading edge genes, overall set NES and FDR were produced for the top 20 positively and negatively enriched gene sets, regardless of statistical significance level.

For dermal RNA-seq data, further and complimentary gene set analysis via protein analysis through evolutionary relationships (PANTHER; version 14.1) statistical enrichment testing (<http://www.pantherdb.org/>) was also employed. An adjusted P-value was calculated using Bonferroni correction, with a statistical significance cut-off of <0.05. The annotated gene sets utilised for this analysis was PANTHER Gene Ontology (GO)-Slim Biological Process. In the context of morphoea being characterised by altered dermal architecture and histology, predicted that great variation between affected and unaffected dermal tissue pairs prompted complimentary gene set analysis via PANTHER statistical enrichment testing was employed, using the annotated PANTHER Gene Ontology (GO)-Slim Biological Process gene sets, to provide comparative validation of dermal RNA-seq GSEA. Compared to the Hallmark gene sets utilised in GSEA, PANTHER GO Biological Processes include a very broad array of interactions and processes and hence can provide useful concurrent analysis.

Finally, STRING online database (https://string-db.org/) version 11 utilised to generate protein-protein network diagrams for products of particular genes of interest from protein coding transcripts generated from RNA-seq. Nodes were connected by a medium confidence level of 0.4 and linked by known evidence of network connections between gene products. In the context of the large datasets in this part of the thesis, disconnected nodes were removed from displays. Nodes were colour coded according to gene set.

***RT-qPCR of selected gene candidates derived from epidermal RNA-seq***

RT-qPCR was utilised to verify randomly selected genes identified from epidermal transcriptomic analysis. Primers were obtained from the Primer Bank databank website (https://pga.mgh.harvard.edu/primerbank/). TBP was used as the housekeeping gene. Quantifast SYBR Green RT-qPCR one-step protocol (Qiagen, Cat ID: 204154) was employed and qPCR assays were performed using a Corbett Rotor Gene 6000 cycler (Qiagen), completed in triplicate. Differences in gene expression were analysed using 2^Ct(housekeeping gene) – Ct(target gene))^.

***Immunohistochemistry of selected genes derived from dermal RNA-seq***

IHC was performed on formalin fixed, wax embedded whole skin sections to verify randomly selected gene candidates of interest identified from dermal transcriptomic analysis. Staining was performed with the Ventana Discovery instrument using the Ventana DAB Map detection kit (760-124). Antigen retrieval protocols were optimised for WNT2 (Abcam: anti-Wnt2/IRP antibody (ab150608)).

Epidermal staining was graded for intensity as low (+), medium (++) or high (+++) and according to approximate percentage of epidermal keratinocytes staining positively (taken as the average of the percentage estimated by three blinded individuals; two dermatopathologists (FD, VS - acknowledgements) and one consultant dermatologist (AMS – first author)). Expression was deemed to be appreciably different if intensity was at least one grade different between morphoea affected and control epidermis, and at least 30% different in density.

Dermal staining was graded for intensity as low (+), medium (++) and high (+++) and according to dispersion of staining. The latter was graded as lightly scattered, moderately scattered or diffusely staining throughout the dermis (fibroblasts and endothelial cells) and this qualitative grading was taken as the average of that estimated by two dermatopathologists (FD and VS - acknowledgements) and one dermatologist (AMS – first author). Expression was deemed to be different if intensity was at least one grade different between morphoea affected and control epidermis, and at least one grade different in density.

**References**

1. Li H. Aligning sequence reads, clone sequences and assembly contigs with BWA-MEM. ArXiv. 2013;1303.

2. Tarasov A, Vilella AJ, Cuppen E, Nijman IJ, Prins P. Sambamba: fast processing of NGS alignment formats. Bioinformatics (Oxford, England). 2015;31(12):2032-4.

3. DePristo MA, Banks E, Poplin R, Garimella KV, Maguire JR, Hartl C, et al. A framework for variation discovery and genotyping using next-generation DNA sequencing data. Nature genetics. 2011;43(5):491-8.

4. McKenna A, Hanna M, Banks E, Sivachenko A, Cibulskis K, Kernytsky A, et al. The Genome Analysis Toolkit: a MapReduce framework for analyzing next-generation DNA sequencing data. Genome research. 2010;20(9):1297-303.

5. Cibulskis K, Lawrence MS, Carter SL, Sivachenko A, Jaffe D, Sougnez C, et al. Sensitive detection of somatic point mutations in impure and heterogeneous cancer samples. Nature biotechnology. 2013;31(3):213-9.

6. Wang K, Li M, Hakonarson H. ANNOVAR: functional annotation of genetic variants from high-throughput sequencing data. Nucleic acids research. 2010;38(16):e164.

7. Kircher M, Witten DM, Jain P, O'Roak BJ, Cooper GM, Shendure J. A general framework for estimating the relative pathogenicity of human genetic variants. Nature genetics. 2014;46(3):310-5.

8. Choi Y, Chan AP. PROVEAN web server: a tool to predict the functional effect of amino acid substitutions and indels. Bioinformatics (Oxford, England). 2015;31(16):2745-7.

9. Choi Y, Sims GE, Murphy S, Miller JR, Chan AP. Predicting the functional effect of amino acid substitutions and indels. PloS one. 2012;7(10):e46688.

10. Adzhubei IA, Schmidt S, Peshkin L, Ramensky VE, Gerasimova A, Bork P, et al. A method and server for predicting damaging missense mutations. Nature methods. 2010;7(4):248-9.

11. Ng PC, Henikoff S. SIFT: Predicting amino acid changes that affect protein function. Nucleic acids research. 2003;31(13):3812-4.

12. Trapnell C, Pachter L, Salzberg SL. TopHat: discovering splice junctions with RNA-Seq. Bioinformatics (Oxford, England). 2009;25(9):1105-11.

13. Wang L, Wang S, Li W. RSeQC: quality control of RNA-seq experiments. Bioinformatics (Oxford, England). 2012;28(16):2184-5.

14. Liao Y, Smyth GK, Shi W. featureCounts: an efficient general purpose program for assigning sequence reads to genomic features. Bioinformatics (Oxford, England). 2014;30(7):923-30.

15. McCarthy DJ, Chen Y, Smyth GK. Differential expression analysis of multifactor RNA-Seq experiments with respect to biological variation. Nucleic acids research. 2012;40(10):4288-97.

16. Liberzon A, Birger C, Thorvaldsdottir H, Ghandi M, Mesirov JP, Tamayo P. The Molecular Signatures Database (MSigDB) hallmark gene set collection. Cell systems. 2015;1(6):417-25.

17. Subramanian A, Tamayo P, Mootha VK, Mukherjee S, Ebert BL, Gillette MA, et al. Gene set enrichment analysis: a knowledge-based approach for interpreting genome-wide expression profiles. Proc Natl Acad Sci U S A. 2005;102(43):15545-50.
